# Could We Safely Avoid a Second Resection in Selected Patients With T1 Non-Muscle-Invasive Bladder Cancer? Preliminary Results of Cost-Effectiveness Study From HUmanitas New Indications for ReTUR (HuNIRe) Multicenter Prospective Trial

**DOI:** 10.3389/fonc.2022.879399

**Published:** 2022-05-18

**Authors:** Roberto Contieri, Giovanni Lughezzani, Nicolò Maria Buffi, Gianluigi Taverna, Alessandro Giacobbe, Emanuele Micheli, Sabato Barra, Piergiuseppe Colombo, Elena Vanni, Giorgio Guazzoni, Massimo Lazzeri, Rodolfo Hurle, HuNIRe Study Group, Paolo Casale

**Affiliations:** ^1^ Department of Biomedical Sciences, Humanitas University, Milan, Italy; ^2^ Department of Urology, IRCCS Humanitas Research Hospital, Milan, Italy; ^3^ Urology Unit, Humanitas Mater Domini, Varese, Italy; ^4^ Struttura Complessa (SC) Urology and Reconstructive Andrology, Humanitas Gradenigo Hospital, Turin, Italy; ^5^ Department of Urology, Humanitas Gavazzeni, Bergamo, Italy; ^6^ Department of Urology, Azienda Socio Sanitarie Territoriali (ASST) Melegnano-Martesana, Milan, Italy; ^7^ Department of Pathology, IRCCS Humanitas Research Hospital, Milan, Italy; ^8^ Business Operations Office, IRCCS Humanitas Research Hospital, Milan, Italy

**Keywords:** second resection, outcome, cystoscopy, urine cytology, non-muscle-invasive bladder cancer

## Abstract

**Objectives:**

The aim of this study is to assess whether restaging transurethral resection (ReTUR) could be safely replaced with urine cytology (UC) and in-office fiexible cystoscopy in selected T1 non-muscle-invasive bladder cancer (NMIBC).

**Materials and Methods:**

This is an ongoing prospective multicenter trial enrolling patients diagnosed with T1 BC from 5 Italian centers. Patients with a macroscopically incomplete initial resection or absence of detrusor muscle were subjected to ReTUR according to European Association of Urology (EAU) guidelines. Conversely, those with a complete tumor resection at initial TUR underwent UC at 3–4 weeks and in-office fiexible white-light and narrow-band cystoscopy at 4–6 weeks. In case of positive UC, or evidence of recurrence at cystoscopy, ReTUR was performed within 2 weeks. Otherwise, patients started Bacillus Calmette–Guérin (BCG) induction course without ReTUR. The primary endpoint was to determine the feasibility and the clinical utility of not performing ReTUR in selected T1 NMIBC patients. The secondary endpoint was to perform a cost–benefit analysis of this alternative approach.

**Results:**

Since May 2020, among 87 patients presenting with T1, 76 patients were enrolled. Nineteen (25%) patients underwent standard ReTUR after initial resection, 10 (13.2%) due to the absence of the detrusor muscle and 9 (11.8%) due to a macroscopically incomplete initial TUR. Overall, 57 (75%) patients initially avoided immediate ReTUR and underwent UC plus in-office flexible cystoscopy. Among them, 38 (66.7%) had no evidence of residual disease and immediately started the BCG induction course. Nineteen patients (33.3%) underwent “salvage” ReTUR due to either positive UC (7; 12.3%) or suspicious cystoscopy (12; 21%). Considering only the patients who initially avoided the ReTUR, disease recurrence was observed in 10/57. The saving of resource for each safely avoided ReTUR was estimated to be 1,759 €. Considering the entire sample, we estimated a saving of 855 € per patient if compared with the EAU guideline approach.

**Conclusion:**

The preliminary results of our trial suggested that ReTUR might be safely avoided in highly selected T1 BC patients with a complete resection at first TUR. Longer follow-up and larger sample size are needed to investigate the long-term oncological outcomes of this alternative approach.

## Introduction

Bladder cancer (BC) is the 10th most diagnosed cancer worldwide, and it is present at least in 75% of the patients as non-muscle-invasive bladder cancer (NMIBC). Transurethral resection (TUR) and subsequent intravesical Bacillus Calmette–Guérin (BCG) course are the standard treatments in high-risk (HR) NMIBC. However, these patients are characterized by a high risk of both recurrence and progression to MIBC. The European Association of Urology (EAU) guidelines suggest performing a second resection [restaging transurethral resection (ReTUR)] before the BCG induction course within 2–6 weeks from the first resection in case of incomplete initial resection, absence of detrusor muscle (DM) in the pathological specimen [with the exception of Ta Low grade (Ta-LG) tumors and primary carcinoma *in situ* (CIS)], or T1 NMIBC (1). ReTUR should remove any residual disease and resample the initial resection area in order to reduce the risk of understaging and the rate of residual disease after first resection (2). Furthermore, according to several authors, ReTUR might decrease recurrence and progression rate while increasing cancer-specific survival (CSS) and overall survival (OS) (3, 4).

However, Gontero et al. (5) suggested that immediate ReTUR may not improve long-term oncological outcomes in those cases where the DM is present at primary TUR, raising concerns about the clinical utility of ReTUR in selected patients. Furthermore, even if considered as a minor surgery, TUR is not a risk-free procedure with a 5% rate of postoperative complications (6). In addition, TUR has a critical impact on the psychological health and quality of life of patients (7).

Finally, BC is defined as having the highest resource consumption on the health system per patient (8). Rationalizing the cost of NMIBC treatment and follow-up may offer the chances to save money and resources (9). Consequently, there is an unmet clinical need to avoid potentially unnecessary endoscopic procedures and save resources without affecting oncological outcomes.

The “HuNIRe” trial tested the hypothesis that avoiding immediate ReTUR in selected patients with T1 NMIBC might be feasible, oncologically safe, and cost-effective.

## Materials and Methods

### Study Design and Population

Data were extracted by a prospective observational multicenter trial enrolling patients diagnosed with T1 NMIBC from a tertiary university hospital as a reference center and 4 additional hospitals of Humanitas Group [HUmanitas New Indication for ReTUR (HuNIRe) trial]. The HuNIRe protocol was approved by the local ethics committees after the approval of the reference center ethics committee (n. 2503 of the 08.05.2020 ICH-010).

Since May 2020, consecutive patients with a pathological diagnosis of T1 NMIBC and aged >18 years old were considered as cases of interest. Patients who had a history of BCG instillations or previous or concomitant upper tract urothelial cancer or those with histological variants were excluded. All patients were informed about the rationale and the purpose of the study and signed a written informed consent. Surgeries were performed by experienced urologists. Experienced urologists were defined as urologists who have performed more than 100 TURs. The completeness of the resections was reported by the surgeon at the end of the resection in a dedicated surgical checklist (**Supplementary Figure S1**).

Patients with a macroscopically incomplete initial resection or in the absence of DM in the histological specimen of primary TUR underwent ReTUR within 2–6 weeks according to EAU guidelines. Alternatively, T1 patients with a macroscopically complete tumor resection at initial TUR underwent urine cytology (UC) after 3–4 weeks and in-office flexible white-light and narrow-band imaging (NBI)-enhanced cystoscopy after 4–6 weeks.

In case of positive UC or evidence of tumor at cystoscopy, ReTUR was performed within 2 weeks. Alternatively, patients started BCG induction course without undergoing ReTUR. Conversely, patients with NMIBC or no residual tumor on ReTUR or those with no visible residual tumor at 4–6-week cystoscopy underwent 6-week induction course with intravesical BCG followed by standard maintenance scheme, which consisted of weekly intravesical BCG instillations for 3 weeks at 3, 6, 12, 18, 24, 30, and 36 months (10).

Follow-up consisted of cystoscopy and urinary cytology at 3 months followed by cystoscopy and cytology every 3 months for a period of 2 years and every 6 months thereafter. All patients underwent computed tomography of the upper urinary tract after the diagnosis and yearly thereafter (1). UC was considered positive when included in diagnostic categories 3–6 of The Paris System for Reporting Urinary Cytology (11). All pathology specimens from TUR were reviewed by a genitourinary pathologist at the reference institution (PC).

### Variable of Interest

The primary endpoint was to determine the feasibility and the clinical utility of avoiding ReTUR in selected T1 NMIBC patients. Clinical utility was defined as the rate of unnecessary ReTUR avoided, while recurrence is considered as the presence of any bladder tumor during the follow-up. The secondary endpoint was to perform a cost–benefit analysis of this alternative approach. Resource consumption analysis was based on the impact on the direct use of resources per patient. No overhead or indirect costs were considered, while costs related to the operation, length of stay, outpatient consumables, and nursing care were included. The average time of use for the operating room, average hospital stays, and diagnostic tests, including specialist visits before and after admission, were estimated from 150 TURs performed in the reference center from December 2019 to May 2020.

### Statistical Analysis

Categorical variables were reported as frequencies and proportions; continuous variables were reported as medians and interquartile ranges (IQRs). For distribution analysis of categorical variables, chi-square test or Pearson’s exact test was used, as pertinent. Mann–Whitney test was used to compare the median of continuous variables with nonparametric distribution.

Kaplan–Meier method was used to estimate recurrence-free survival (RFS). All statistical analyses were performed with the Stata/SE, version 17 (Stata Corp. LP, College Station, TX, USA).

## Results

**Figure 1:**
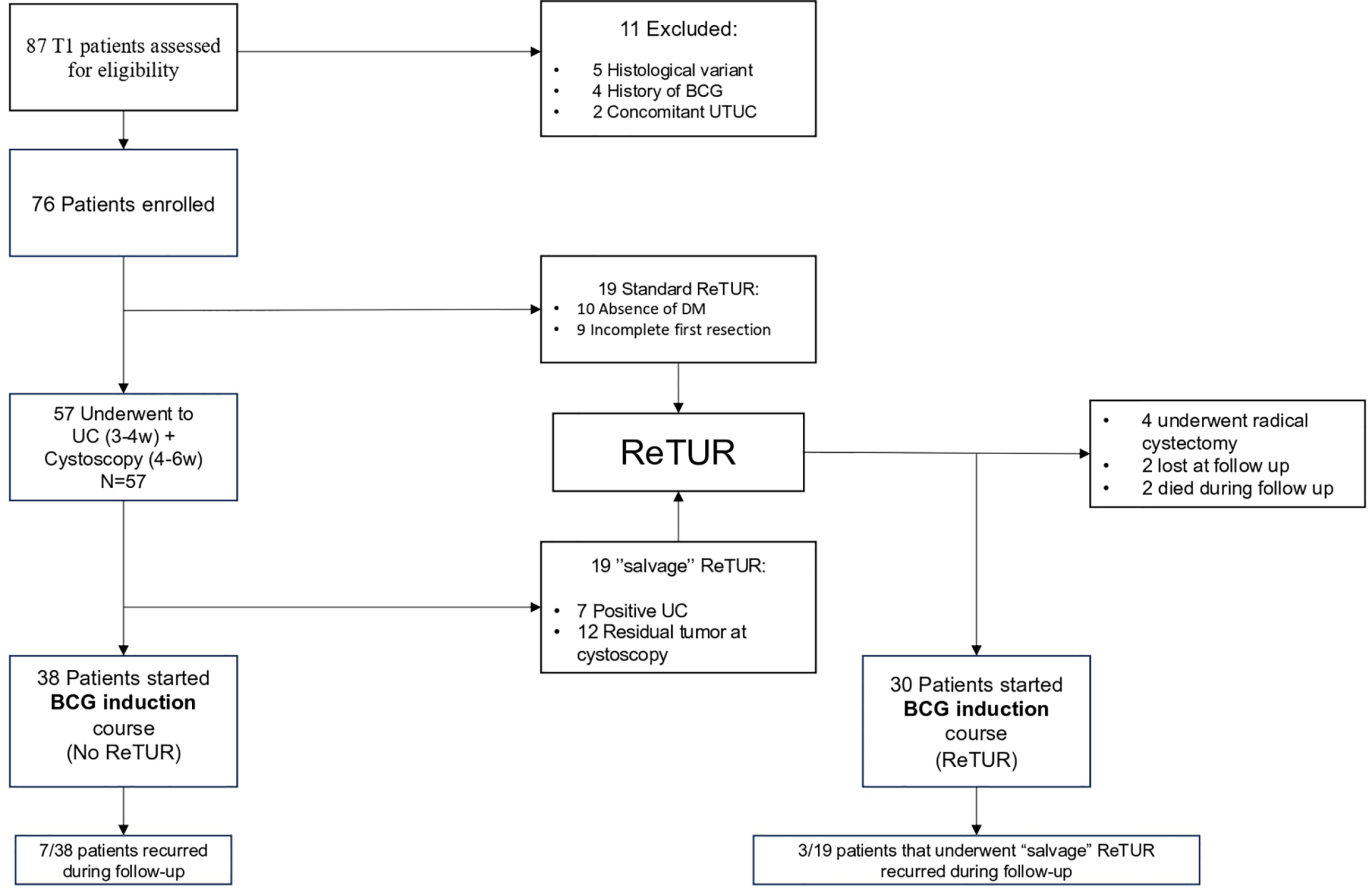
Flow of patients through the study. NMIBC, Non-Muscle Invasive Bladder Cancer; MIBC, Muscle Invasive Bladder Cancer; DM, Detrusor Muscle; UC, Urine cytology; UTUC, Upper urinary tract urothelial cancer.

Among 87 patients with T1 NMIBC presenting between May 2020 and December 2021, 76 patients met the inclusion criteria, signed the informed consent, and were subsequently enrolled in the trial. Median age was 75.4 years (67.6–80.3) with the majority being men (n = 63; 82.9%). Most of the patients presented with primary BC (n = 68; 89.4%), while 8 patients (10.6%) had a recurrent BC with a previous diagnosis of G2/LG Ta BC in 4 patients and G1/LG Ta BC in the other 4 cases; none of the patients had a history of CIS. Median time to recurrence was 31 months (IQR 22.5–32.7), and 3 patients had previous chemotherapy instillations with mitomycin. Multifocal lesions and tumor greater than 3 cm in diameter were observed in 18 (23.7%) and 33 (43.5%) patients, respectively. Only 6 patients (7.9%) had a low-grade NMIBC. Patient characteristics are summarized in **Table 1**, while **Table 2** shows patient characteristics stratified by prior recurrence status. Nineteen (25%) patients underwent standard ReTUR after initial resection, 10 (13.2%) due to the absence of the DM and 9 (11.8%) due to a macroscopically incomplete initial TUR. Residual disease at ReTUR was found in 11 patients (57.9%); among them, one patient was upstaged to MIBC, 5 patients presented residual high-grade (HG) T1 BC, 3 had residual CIS, while only 2 patients were diagnosed with LG Ta BC. Overall, 57 (75%) patients initially avoided immediate ReTUR and underwent UC plus in-office flexible cystoscopy. Among them, 38 (66.7%) had no evidence of residual disease and immediately started the BCG induction course. On the other hand, 19 patients (33.3%) underwent “salvage” ReTUR due to either positive UC (7; 12.3%) or suspicious cystoscopy (12; 21%); histological results of ReTUR are shown in **Table 3**. The complete workflow of enrolled patients in the study is depicted in **Figure 1**.

**Table 1:** Characteristics of the patients.

		Overall population
		N = 76
Follow-up in months, median (IQR)		11 (8.1–15)
Age, median (IQR)		75.4 (67.6–80.3)
Gender, n (%)	Men	63 (82.9)
	Women	14 (17.2)
Primary tumor, n (%)		68 (89.4)
Tumor size, n (%)	< 3cm	43 (56.5)
	> 3cm	33 (43.5)
Multifocal tumor, n (%)		18 (23.7)
Associated CIS, n (%)		6 (7.8)
Primary grade (WHO 1973)	G2	11 (14.5)
	G3	65 (85.5)
Primary grade (WHO 2004/2016)	LG	6 (7.9)
	HG	70 (92.1)
Initial TUR, n (%)	Complete	67 (88.1)
	Incomplete	9 (11.9)
DM absence, n (%)		10 (13.1)
Standard ReTUR initially avoided, n/N (%)		57/76 (75)
Salvage ReTUR	Positive UC	7/57 (12.3)
(Reason) n/N (%)	Positive cystoscopy	12/57 (21.1)

IQR, interquartile range; ReTUR, second resection; salvage ReTUR, second resection after urine cytology + cystoscopy (as per HuNIRe protocol); LG, low grade; HG, high grade; DM, detrusor muscle.

**Table 2:** Characteristics of the patients stratified by prior recurrence status (primary vs. recurrent).

		Primary BC (n = 68)	Recurrent BC (n = 8)	p-value
Age, median (IQR)		74.7 (65.6–80.4)	76.1 (70.8–78.3)	0.749
Gender, n (%)	Men	57 (83.8)	5 (62.5)	
	Women	11 (16.2)	3 (37.5)	0.141
Tumor size, n (%)	<3 cm	35 (51.5)	8 (100)	
	>3 cm	33 (48.5)	0 (0)	0.009
Multifocal tumor, n (%)		15 (22.1)	3 (37.5)	0.331
Associated CIS, n (%)		5 (7.8)	1 (12.5)	0.610
Primary grade (WHO 1973)	G2	10 (14.7)	1 (12.5)	
	G3	58 (85.3)	7 (87.5)	0.867
Primary grade (WHO 2004/2016)	LG	6 (8.8)	0 (0)	
	HG	62 (91.2)	8 (100)	0.381
Initial TUR, n (%)	Complete	61 (89.7)	6 (75)	
	Incomplete	7 (10.3)	2 (25)	0.223
DM absence n (%)		10 (14.7)	0 (0)	0.244

IQR, interquartile range; LG, low grade; HG, high grade; DM, detrusor muscle.

**Table 3:** Histopathology result at ReTUR for the 19 patients who underwent a second resection because of either positive UC or cystoscopy and for the 10 patients who had recurrence during the follow-up.

	Final pathology	N = 19
“salvage” ReTUR, n (%)	Negative	3 (15.8)
	LG Ta	1 (5.3)
	HG Ta	5 (26.3)
	HG T1	3 (15.8)
	CIS	7 (36.8)
	Final pathology	N = 10
Recurrence during follow-up, n (%)	LG Ta	2 (20)
	HG Ta	3 (30)
	HG T1	3 (30)
	CIS	2 (20)

LG, low grade; HG, high grade; CIS, carcinoma in situ.

Only 1 upstaging to MIBC at standard ReTUR was observed, while two patients died during the follow-up from causes other than BC. Considering only the 57 patients who initially avoided the ReTUR, with a median follow-up of 11 months, disease recurrence was observed in 10/57; among them, 7 belonged to the group that avoided ReTUR and underwent directly a BCG induction course, while the other 3 initially avoided ReTUR but underwent salvage ReTUR because of positive UC or cystoscopy. Notably, 8 patients had an HG recurrence (**Table 2**). RFS rates were 95.2% and 74.3% at 6 and 12 months, respectively. Furthermore, none of the patients progressed to MIBC.

The mean cost of ReTUR was estimated at 1,854 € per patient, while the mean cost of conservative approach (UC and outpatient cystoscopy) was estimated at 95 € per patient. Resource consumption and related cost analysis are shown in **Table 4**. The estimated resource saving for each avoided ReTUR was 1,759 €. Considering a ReTUR rate of 50% (38/76) in the whole sample, savings of 48.6% per patient were estimated when compared with the EAU guideline approach.

**Table 4:** Resource consumption and related cost analysis.

Standard management		Conservative management (Cystoscopy + UC)	
Variable clinical resources	Related costs (€)	Variable clinical resources	Related costs (€)
Pre-hospital admission evaluation	128.7	Outpatient slot	20
Diagnostic exams during hospital stay (histology included)	243.4	Nursing care for slot	25
Post hospitalization exams	35.1	Medicines and materials	50
		**Total**	95
OR related cost (anesthesiologic and nursing care included)	511.7		
Hospitalization cost (Nursing care included)	626.9		
Medicines and materials	307.8		
**Total**	1,853.6		

UC, urine cytology; OR, operating room.

Our preliminary results show that ReTUR might be avoided in selected T1 BC patients, turning into an estimated savings of about 855.6 € per patient diagnosed with pT1 BC.

## Discussion

Herein, we present the preliminary results of HuNIRe trial; the study was designed to evaluate the possibility of identifying those T1 NMIBC patients who could avoid a second resection and directly begin the BCG induction course.

To the best of our knowledge, this is the first study investigating an alternative approach to standard ReTUR in this subset of patients. According to our preliminary results, ReTUR may be spared in a substantial proportion of appropriately selected patients, and this approach may be effective both from an oncological and from a resource–consumption perspective. More specifically, we avoided 66% of ReTUR in patients with a complete primary resection and, even more importantly, no case of MIBC was missed.

The rationale for avoiding a ReTUR is based on the fact that this approach may be considered an overtreatment in selected individuals and could also lead to a significant delay of adjuvant therapies. The influence of the time between surgical treatment and BCG induction cycle on oncological results is still a matter of debate. However, EAU guidelines recommend the onset of BCG immunotherapy at least 2 weeks after TUR and no time limit is set (1). Performing a ReTUR in all patients may significantly delay the start of the BCG course, which could be related to worse survival outcomes (12).

A recent randomized trial showed that patients subjected to a second TUR had significantly higher recurrence-free, progression-free, and overall survival (13). However, this study included only the use of intravesical chemotherapy as adjuvant therapy, whereas adjuvant BCG instillations are known to be superior for preventing recurrence and progression in NMIBC and are the current standard of care according to EAU guidelines (14, 15).

Although there has been a softening of the European guideline position regarding ReTUR, which is no longer indicated for HG Ta NMIBC patients, there is still an open debate about its clinical usefulness in every patient with a T1 BC (16).

Recently, some authors found that the presence of DM in TUR specimen, the absence of CIS, and resection performed with *en bloc* technique were independent predictors of the absence of tumor at ReTUR, suggesting the possibility of avoiding ReTUR in selected patients (17, 18). Furthermore, in a multicenter retrospective cohort study including HG T1 NMIBC patients treated with BCG, Gontero et al. (5) showed that ReTUR was associated with superior oncological outcomes, only in case of the absence of DM in the surgical specimen. Nevertheless, these results were limited by the retrospective design of the study and the low rate of ReTUR performed.

Another debated issue concerns the risk of upstaging to MIBC at ReTUR that ranges from 0% to 45% (19). A recent systematic review of the literature found a negligible risk of upstaging in many series (1−4%), thus underscoring a possible effect of surgeons’ experience on primary TUR outcomes (20).

Although the risk of upstaging is non-negligible, the rationale of the study is based on the strong belief that a “good quality” TUR could minimize this risk. Herr et al. (21) stated that a well-performed TUR is one of the most effective and powerful procedures at the disposal of the urologist. In this regard, Mostafid et al. (22) reported a list of optimal best practices to adopt to optimize quality and outcomes of TUR.

While the presence of DM in the pathological specimen is considered as a surrogate of TUR quality and is related to a lower residual disease rate at ReTUR (19, 23), this factor alone is probably not enough to guide clinical decisions. Therefore, both cystoscopy and UC were also included in the study workflow.

Cystoscopy was performed from 4 to 6 weeks after TUR to detect any residual disease; a controversial point may be the presence of fibrin covering the resection scar, although after this time, it should not prevent residual disease from being visualized. Furthermore, all cystoscopies were performed with NBI, which was shown to be superior to white light alone in terms of BC detection (24). However, while NBI represents a useful tool in the diagnosis of BC, its availability is restricted, limiting the reproducibility of the study.

Likewise, all patients underwent UC at 3–4 weeks to detect malignant cells that could be related to residual disease in the bladder. The introduction of the new Paris classification has improved both UC sensitivity (SE), ranging from 34% to 95%, and negative predictive value (NPV), ranging from 46% to 86% in HG BC. However, specificity (SP) remains lower than 70% (25). Through the last decades, numerous molecular urine markers for diagnosis of BC have been developed (26, 27); it is conceivable that one of these markers, combined with cystoscopy and UC, could be used in the future to improve the identification of those patients who can avoid ReTUR.

One of the strengths of our study is the analysis of the impact that avoiding a ReTUR in selected T1 NMIBC patients might have on hospital resource.

However, the impact of an NMIBC diagnosis extends beyond quantifiable factors. Indirect costs, such as days of work lost by the patient and his/her caregivers, are difficult to estimate and would deserve a detailed analysis (8). Indeed, our analysis only considers resource consumption, which is a “direct” and immediate measure of saving for the hospital. Nevertheless, according to Value-Based Healthcare logic (28), for each intervention avoided, the hospital can invest the saved resources in new therapies or to treat other patients.

Lastly, Ferro et al. (29) reported in a recent study that the time to ReTUR was significantly increased during the severe acute respiratory syndrome coronavirus 2 (SARS-CoV-2) pandemic; this finding suggest that an outpatient management, which in many cases was preserved during the pandemic, would not have resulted in delayed treatment.

Our study is not devoid of limitations mainly due to the lack of a control group. We also acknowledge that the small sample size could have limited the strength and reproducibility of our results. Additionally, the median follow-up was limited to 11 months, therefore being too short to provide an accurate estimate of oncological outcomes. Indeed, a comparison of long-term oncological outcomes between patients enrolled in the current protocol and those subjected to the standard of care is warranted.

## Conclusions

In the current prospective study, we demonstrated that avoiding ReTUR in appropriately selected patients with T1 NMIBC is feasible and safe. This approach may limit the psychological impact and potential morbidities of a second surgery while providing significant resource savings.

However, the findings are preliminary and internally validated; furthermore, the study design does not permit to conclude on the oncological safety of this approach. Therefore, in order to confirm our preliminary results, randomized controlled trials are mandatory.

Furthermore, extended follow-up and a larger sample size, as well as further studies on novel diagnostic tools, are needed to obtain a better patient selection and a safer therapeutic strategy balancing risks, oncological outcomes, and resource consumption.

## Data Availability Statement

The original contributions presented in the study are included in the article/**Supplementary Material**. Further inquiries can be directed to the corresponding author.

## Ethics Statement

The studies involving human participants were reviewed and approved by Comitato etico IRCCS Humanitas Research Hospital. The patients/participants provided their written informed consent to participate in this study.

## HuNIRe Study Group

**Paolo Casale**, IRCCS Humanitas Research Hospital Rozzano, Milan, Italy; **Alberto Saita**, IRCCS Humanitas Research Hospital Rozzano, Milan, Italy; **Andrea Gobbo**, Department of Biomedical Sciences, Humanitas University Pieve Emanuele, Milan, Italy; **Edoardo Beatrici**, Department of Biomedical Sciences,Humanitas University Pieve Emanuele, Milan, Italy; **Pier Paolo Avolio**, Department of Biomedical Sciences, Humanitas University Pieve Emanuele, Milan, Italy; **Alessandro Uleri**, Department of Biomedical Sciences, Humanitas University Pieve Emanuele, Milan, Italy; **Marco Paciotti**, Department of Biomedical Sciences, Humanitas University Pieve Emanuele, Milan, Italy; **Vittorio Fasulo**, Department of Biomedical Sciences, Humanitas University Pieve Emanuele, Milan, Italy; **Nicola Frego**, Department of Biomedical Sciences, Humanitas University Pieve Emanuele, Milan, Italy; **Davide Maffei**, Department of Biomedical Sciences, Humanitas University Pieve Emanuele, Milan, Italy; **Pietro Diana**, Department of Biomedical Sciences, Humanitas University Pieve Emanuele, Milan, Italy; **Matteo Zanoni**, Urology Unit, Humanitas Mater Domini, Castellanza, Varese, Italy; **Luigi Domanico**, Department of Urology, Humanitas Gavazzeni, Bergamo,Italy; **Devis Collura**, SC Urology and Reconstructive Andrology PO Humanitas Gradenigo, Turin, Italy; **Maria Grazia Elefante**, Department of Pathology, IRCCS Humanitas Research Hospital, Rozzano, Milan, Italy; **Miriam Cieri**, Department of Pathology, IRCCS Humanitas Research Hospital, Rozzano, Milan, Italy.

## Author Contributions

RC, RH, and GG contributed to conception and design of the study. RC organized the database. RC and GL performed the statistical analysis. RC, RH, ML, and GL wrote the article. All authors contributed to article revision and read and approved the submitted version.

## Conflict of Interest

The authors declare that the research was conducted in the absence of any commercial or financial relationships that could be construed as a potential conflict of interest.

## Publisher’s Note

All claims expressed in this article are solely those of the authors and do not necessarily represent those of their affiliated organizations, or those of the publisher, the editors and the reviewers. Any product that may be evaluated in this article, or claim that may be made by its manufacturer, is not guaranteed or endorsed by the publisher.
