# Supplementary material for: Could We Safely Avoid a Second Resection in Selected Patients With T1 Non-Muscle-Invasive Bladder Cancer? Preliminary Results of Cost-Effectiveness Study From HUmanitas New Indications for ReTUR (HuNIRe) Multicenter Prospective Trial
Source: Front Oncol. 2022 May 18;12:879399. doi: 10.3389/fonc.2022.879399 (PMC9157494; doi:10.3389/fonc.2022.879399)
Supplement: Supplementary file 1 [file Table_1.docx]

Supplementary Material

**Supplementary Figure 1.** Surgical checklist filled in at the end of the procedure by the surgeon
